# The effects of exercise and cold exposure on mitochondrial biogenesis in skeletal muscle and white adipose tissue

**DOI:** 10.20463/jenb.2017.0020

**Published:** 2017-06-30

**Authors:** Nana Chung, Jonghoon Park, Kiwon Lim

**Affiliations:** 1. Physical Activity & Performance Institute (PAPI), Konkuk University, Seoul Republic of Korea; 2. Department of Physical Education, Korea University, Seoul Republic of Korea; 3. Department of Physical Education, Konkuk University, Seoul Republic of Korea

**Keywords:** Exercise, Cold exposure, PGC-1α, NRF-1, Tfam, Mitochondrial biogenesis

## Abstract

**[Purpose]:**

The purpose of this study was to determine whether exercise or/and cold exposure regulate mitochondria biogenesis-related gene expression in soleus and inguinal adipose tissue of mice.

**[Methods]:**

Forty ICR 5-week old male mice were divided into four groups: thermoneutrality-untrained (23 ± 1 °C in room temperature, n=10), cold-water immersion (24 ± 1 °C, n=10), exercise in neutral temperature (34 ± 1 °C, n=10), and exercise in cold temperature (24 ± 1 °C, n=10). The mice performed swimming exercise (30 min to 60 min, 5 times) for 8 weeks. After 8 weeks, we confirmed mitochondrial biogenesis-related gene expression changes for peroxisome proliferator-activated receptor gamma coactivator-1 alpha (PGC-1α), nuclear respiratory factors 1 (NRF1), and mitochondrial transcription factor A (Tfam) in soleus muscle and inguinal adipose tissue, and the related protein expression in soleus muscle.

**[Results]:**

In soleus muscle, PGC-1α expression significantly increased in response to cold exposure (p = 0.006) and exercise (p = 0.05). There was also significant interaction between exercise and cold exposure (p = 0.005). Only exercise had a significant effect on NRF1 relative expression (p=0.001). Neither cold exposure nor the interaction showed significant effects (p = 0.1222 and p = 0.875, respectively). Relative Tfam expression did not show any significant effect from exercise. In inguinal adipose tissue, relative PGC-1α expression did not significantly change in any group. NRF1 expression showed a significant change from exercise (p = 0.01) and cold exposure (p = 0.011). There was also a significant interaction between exercise and cold exposure (p = 0.000). Tfam mRNA expression showed a significant effect from exercise (p=0.000) and an interaction between exercise and cold exposure (p=0.001). Only temperature significantly affected PGC-1α protein levels (p=0.045). Neither exercise nor the interaction were significant (p = 0.397 and p = 0.292, respectively). NRF1 protein levels did not show a significant effect in any experimental treatments. Tfam protein levels showed a significant effect in the exercise group (p=0.012), but effects of neither cold exposure nor the interaction were significant (p = 0.085 and p=0.374, respectively).

**[Conclusion]:**

Exercise and cold exposure promoted increased expression of mitochondrial biogenesis- related genes in soleus muscle. Only cold exposure had a significant effect on PGC-1α protein expression and only exercise had a significant effect on Tfam protein expression. In inguinal adipose tissue, there was interaction between exercise and cold exposure in expression of mitochondrial biogenesis-related genes.

## INTRODUCTION

Recently, mitochondrial dysfunction has been recognized as an important contributor to an array of human pathologies^1- 5^. A variety of ‘mitochondrial medicine’ treatments have been evaluated by randomized clinical trials; unfortunately, none have delivered breakthrough results. In addition, long-term pharmaceutical use has been limited by lack of efficacy, poor long-term adherence rates, and adverse effects^6^. On the other hand, environmental stresses including exercise and cold exposure, which increase cellular energy demand, act to trigger mitochondrial biogenesis^7-10^. Numerous studies have shown that exercise or cold exposure increases peroxisome proliferator-activated receptor gamma coactivator-1 alpha (PGC-1α) expression in skeletal muscle and adipose tissue^8-12^. PGC-1α is the key regulator of mitochondrial biogenesis. PGC-1α interacts with nuclear respiratory factor-1 (NRF-1), which activates mitochondrial transcription factor A (Tfam), thus activating the coordinated expression of mitochondrial proteins^13,14^. Exercise increases cellular energy demand, which increases intracellular AMP, Ca2+ concentrations, free phosphate groups (Pi), and reactive oxygen species (ROS)^15^. These substances are potent signaling transducers and activate calcium/calmodulin-dependent protein kinases (CaMK), AMP-activated protein kinase (AMPK), and p38 mitogen-activated kinase (p38MAPK), which trigger transcription and activation of PGC-1α and mitochondrial biogenesis^16^. However, the sensory nerves in peripheral tissues first sense cold. This information is then received and processed in the hypothalamus, which controls the activity of the sympathetic nervous system (SNS) leading to the release of adrenergic hormone onto fat cells. Adrenergic stimuli activate G protein-coupled β-adrenergic receptors. This stimulates cell proliferation. There are multiple changes within the adipose tissue, particularly increased mitochondrial content^17, 18^. However, it is unclear whether exercise and cold exposure act independently or dependently, because previous studies have reported only the effect of exercise^6, 8, 16^ or cold exposure^9-19^. In addition, whether cold and exercise act through a common mechanism or if different mechanisms lead to a common phenotype is unclear. Hence, the purpose of this study is to survey mitochondrial related gene responses to exercise and cold exposure and to determine whether exercise and cold exposure interact to modulate mitochondrial biogenesis in soleus muscle and inguinal adipose tissue, because soleus muscle and inguinal adipose tissue have a great capacity for adaptive responses to external stimuli such as cold and exercise. 

## METHODS

### Animals and treatments

Male ICR mice (n=40) were purchased from Orient Bio Company (Seongnam, Korea) at five weeks of age. All mice were kept in a specific pathogen-free (SPF) environment (humidity 50%, temperature 23 ± 1 °C), housed in conventional cages (n=5 per cage) on a 12-h:12-h light/dark cycle with free access to water, and provided non-purified commercial diet (5L79, Orient Bio Inc., Seongnam, Korea). After 1 week of adaptation, they were randomly divided into four groups: thermoneutrality-untrained (CON, 23 ± 1 °C in room temperature, n=10), cold water immersion (CWI, 24 ± 1 °C, n=10), exercise in neutral temperature (ENW, 34 ± 1 °C, n=10), and exercise in cold temperature (ECW, 24 ± 1 °C, n=10). The body weights and food intake were monitored daily. 

All experimental procedures were approved by the Animal Experiment Research Center of Konkuk University and Ethics Committee of the Konkuk University Institutional Animal Care and Use Committee (Permit Number: KU14098). 

### Experimental design

It is difficult to fully control the variables in these experiments when the environmental temperature is set low because the animals have hair and the heat generated during the training may change the internal temperature of the animal training chamber. Swimming training induces PGC-1α protein expression, suggesting that this exercise is a potent stimulus for mitochondrial biogenesis^19-21^. Thus, we adopted a swimming exercise program to evaluate the effects of exercise at different temperatures on mitochondrial biogenesis. Mice were trained to swim using a modified protocol from Kang & Kim (2015)^19^. Swim training commenced at specific times with a frequency of 5 times per week for 8 weeks. The water bath was 66 cm in width, 38 cm in depth, and 32 cm in height. The water temperature was 34 ± 1 °C for the neutral temperature group and 24 ± 1 °C for the cold-water groups. Trained mice (n=20) were first introduced to swimming for 3 days. After the adaptation period for swimming, mice were randomly divided in to two groups: ENW, n=10 and ECW, n=10. For the next 4 weeks, they were trained to swim for 30 min, which was progressively increased to 60 min. During the last 4 weeks of the training program, mice were trained for 60 min with progressively increasing loads. Beginning in the 5th week of the experimental period, we measured the weight on the first day of each week and put a metal tip weight (increasing from 1 to 4% of mouse weight) on each mouse tail. 

**Figure 1.**
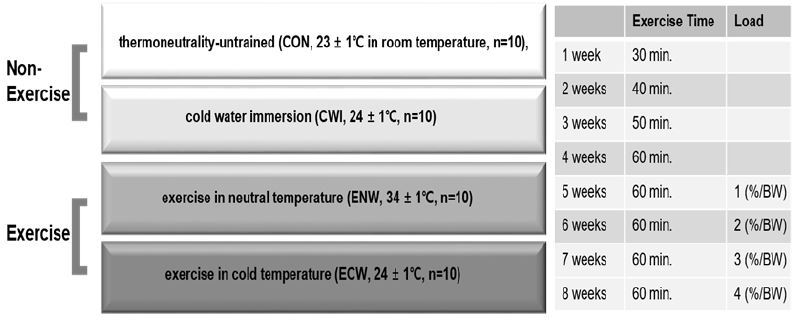
Experimental design and training program.

CWI mice were immersed in a water bath filled with cold water at shoulder height. Similar to the exercise groups, the CWI group was immersed in cold water for 30 min, which was gradually increased to 60 min for the first 4 weeks, and this training time was maintained until the end of the experiment without load. The CON animals remained sedentary in their cages for the duration of the 8-week training program. Details of the exercise program are shown in Fig. 1. 

### Collection of tissue samples

At the end of the experimental protocol, all the animals were fasted overnight with free access to water. Mice were anesthetized with avertin and the soleus muscle and inguinal adipose tissue were dissected rapidly. The isolated tissues were frozen in liquid nitrogen and stored at −80 °C until analysis. 

### Total RNA extraction and reverse transcription

Total RNA from soleus muscle and inguinal adipose tissue were extracted using Trizol reagent (Life Technologies Inc.) in accordance with the manufacturer’s protocol. Briefly, tissue was homogenized (Qiagen, TissueRuper, Hilden, Germany) with 1 ml Trizol reagent for 5 min at room temperature. Then, 200 μl chloroform was added to the tubes, which were centrifuged for 15 min at 12,000 g. The supernatant was transferred to another tube with isopropanol (Iso-Propyl Alcohol, Duksan Pure Chemicals, Korea). The RNA was pelleted by centrifugation (15 min at 12,000 g) with diluted DEPC 75% ethanol and air-dried. RNA pellets were diluted in 30 μl DEPC water and heated at 55 °C for 10 min. The RNA was quantified using a nano block measuring absorbance at 260 nm (Multiskan Go micro reader, ThermoFischer, CA, USA) and the RNA purity was assessed using the 260/280 nm ratio. cDNA synthesis using the RNA was performed according to the manufacturer’s protocol (cDNA synthesis master mix, GenDEPOT, Barker, TX, USA). The gene expression was analyzed using the manufacturer’s protocol (amfiEco Taq DNA Polymerase, GenDEPOT). The primer sequences are in Table 1. PCR products were assessed on a 10% agarose (Sigma Aldrich, Germany) gel in 1X TAE buffer (Tris-Acetate-EDTA) with DNA gel staining solution (safe pinky DNA gel staining solution (10,000X), GenDEPOT). PCR product bands were measured with Printgraph 2M (ATTO Biotechnology, Sungnam, Korea). 

**Table 1.** Primer sequences for RT-PCR.

Target mRNA	Forward primer	Reverse primer
GAPDH	5’-AAC TTT GGC ATT GTG GAA GG-3’	5’-ACA CAT TGG GGG TAG GAA CA-3’
PGC-1α	5’-TCC CGA AGA CAC TAC AGG TT-3’	5’-AAG GAG CCA CTG AAC ACA CT-3’
NRF-1	5’-ACG TTA CAG GGC GGT GAA AT-3’	5’-GCT GTC CGA TAT CCT GGT GG-3’
Tfam	5’-TAG GCA CCG TAT TGC GTG AG-3’	5’-CCA CAG GGC TGC AAT TTT CC-3’

### Western blot analysis

Soleus muscle and inguinal adipose tissue were homogenized in homogenization buffer (1 ml RIPA Lysis, 10 μl Protease Inhibitor, 10 μl Phosphatase Inhibitor per sample; EzRIPA Lysis, ATTO) and homogenizer (Qiagen, TissueRuper). Homogenates were centrifuged at 4 °C and 11,463 g for 15 min. The supernatant protein concentration was determined with GenDEPOT Protein Assay Plus Reagent (GenDepot) using the Bradford method with bovine serum albumin (BSA) as the standard. The samples were boiled at 100 °C for 5 min. For each sample, total protein (30 μg) was resolved by 12% sodium dodecylsulfate-polyacrylamide gel electrophoresis and transferred to polyvinylidene fluoride (PVDF) membranes (Millipore, Billerica, MA, USA) at 100 V for 2 h. The membrane was blocked for 1 h with 5% BSA solution and then washed 3 times (5, 5, and 10 min) with Tris-buffered saline – Tween 20 (TBS-T) buffer. After overnight incubation at 4 °C with primary antibodies (PGC-1α (H-300), NRF1 (H-285) (Santa Cruz Biotechnology, USA), and Tfam (ab138351) (Abcam, UK), dilution 1:1000 in a 5% BSA solution), the membrane was washed with TBS-T buffer and incubated with donkey anti-rabbit IgG H&L (HRP) (ab6802, Abcam, UK, dilution 1:1000) at room temperature for 1 h. Immunodetection was carried out with ECL detection reagent (GE healthcare, UK). The image was obtained on an imager (LAS-2000, GE healthcare Life science, UK). The protein amount was analyzed using ImageJ software (National Institutes of Health, USA). 

### Statistical analysis

Statistical analyses were performed with SPSS 23.0 for windows (IBM Corp., Armonk, USA). Values are means ± SE (standard error) for the indicated number of experiments. The two-way analysis of variance method was applied to determine the interaction and main effect with exercise and temperature. A Tukey post-hoc analysis was conducted if a significant interaction or main effect were obtained. A priori, the level of significance was set at 0.05. 

## RESULTS

### Body weight and food intake changes

To clarify the effect of exercise or/and cold exposure on basic metabolism in mice, we compared the body weight and daily food intake of mice from cold exposed (cold) or neutral temperature (neutral) and exercised or non-exercised for 8 weeks. 

**Figure 2.**
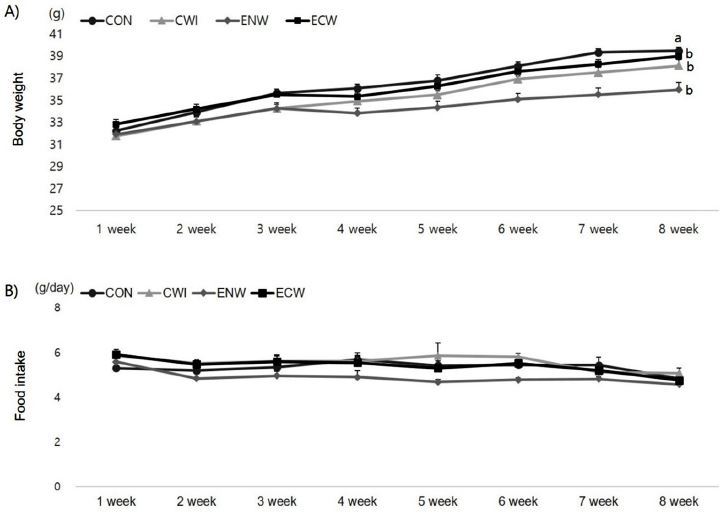
Effects of exercise or/and cold exposure on body weight and food intake. (A) Body weight (B) Food intake. Thermoneutrality-untrained (CON); cold water immersion (CWI); exercise in neutral temperature (ENW); exercise in cold exposure (ECW); Data are presented as means ± SE (n=8 per group). Different letters indicate significant differences (p < 0.05) between groups.

Figure 2 shows the results for mouse body weight and daily food intake variation for 8 weeks. At the start of the study, there were no differences in body weight between the four groups. The body weight of mice increased progressively throughout the study, and final body weight was significantly changed in the exercise group (0.011). In addition, a significant interaction between exercise and cold exposure (p=0.000) was found. Thus, animals that exercised in neutral temperature water had a lower body weight than animals used as controls, those immersed in cold water, and those exercised in cold water. The final body weight of the mice trained in neutral temperature water was 10% lower than the control group. In addition, cold exposed mice displayed significantly higher food intake than their counterparts at neutral temperature. 

### Soleus muscle and inguinal adipose tissue weight changes

Next, we analyzed whether exercise and cold exposure caused differences in soleus muscle and inguinal adipose tissue weight. At the end of the experimental period, exercise (p = 0.004) and cold exposure (p=0.033) had a significant effect on the ratio of soleus muscle weight to body weight and there was significant interaction between exercise and cold exposure (p = 0.025). The post-hoc test examining group differences was high in the control group compared to other treatment groups. In inguinal adipose tissue, tissue weight showed a significant effect from cold exposure (p=0.000) and interaction (p=0.006), but there was no effect from exercise (p=0.749). 

**Figure 3.**
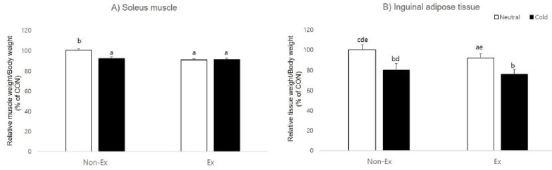
Effects of exercise or/and cold exposure on the soleus muscle and inguinal adipose tissue weight. (A) Ratio of soleus muscle weight to body weight at 8 weeks. (B) Ratio of inguinal adipose tissue weight to body weight at 8 weeks. Two-way ANOVA results: (A) temperature: 0.033, exercise: 0.004, interaction 0.025 (B) temperature: 0.000, exercise: 0.749, interaction 0.006. Data are presented as means ± SE (n=8 per group). Different letters indicate significant differences (p < 0.05) between groups.

### Gene expression

Given that exercise or/and cold exposure changed the mass of muscle and adipose tissue, we investigated the changes in soleus muscle and inguinal adipose tissue gene expression. The potential molecular mechanisms that govern exercise- and cold-induced mitochondrial biogenesis quantified were the transcription factors PGC-1α, NRF1, and Tfam. Gene expression changes between tissues were determined and relative mRNA band density values were calculated as the ratio of gene of interest to that of GAPDH. The data in the control group have been set to 100% for each tissue. 

**Figure 4.**
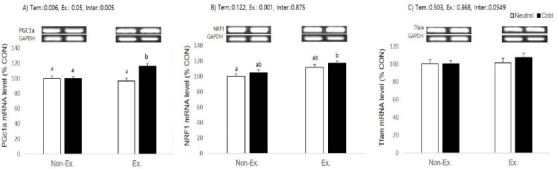
Effects of exercise or/and cold exposure on expression of mitochondrial biogenesis-related genes in the soleus muscle. Data are presented as means ± SE (n=8 per group). (A) peroxisome proliferator-activated receptor gamma coactivator-1 alpha (PGC-1α); (B) nuclear respiratory factor 1 (NRF1); and (C) mitochondrial transcription factor A (Tfam) relative to the expression level of glyceraldehyde 3-phosphate dehydrogenase (GAPDH). Two-way ANOVA results: (A) temperature: 0.006, exercise: 0.05, interaction 0.005 (B) temperature: 0.122, exercise: 0.001, interaction 0.875 (C) temperature: 0.503, exercise: 0.368, interaction 0.0549. Different letters indicate significant differences (p < 0.05) between groups.

In soleus muscle, PGC-1α mRNA levels were significantly increased in the cold exposure ( p= 0.006) and exercise (p = 0.05) groups, and there was a significant interaction between exercise and cold exposure (p = 0.005). Post-hoc analyses for PGC-1α showed exercise or cold exposure alone had no effect, but exercise in cold water induced a higher PGC-1α expression than in other groups. Only exercise had a significant effect on NRF1 relative expression (p=0.001), and neither cold exposure nor the interaction was significant (p = 0.1222 and p = 0.875, respectively). Post-hoc analyses for NRF1 showed that only cold-water exercised animals had a higher expression than the control group. Relative expression of Tfam did not show any significant effect from cold exposure, exercise, or an interaction (p = 0.0549). 

**Figure 5.**
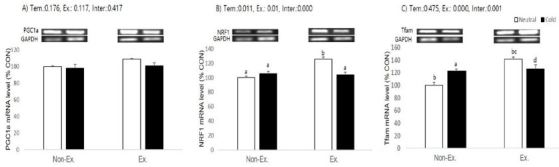
Effects of exercise or/and cold exposure on expression of mitochondrial biogenesis-related genes in the inguinal adipose tissue. Data are presented as means ± SE (n=8 per group). (A) peroxisome proliferator-activated receptor gamma coactivator-1 alpha (PGC-1α); (B) nuclear respiratory factor 1 (NRF1); and (C) mitochondrial transcription factor A (Tfam) relative to the expression level of glyceraldehyde 3-phosphate dehydrogenase (GAPDH). Two-way ANOVA results: (A) temperature: 0.176, exercise: 0.117, interaction 0.417 (B) temperature: 0.011, exercise: 0.01, interaction 0.000 (C) temperature: 0.475, exercise: 0.000, interaction 0.001. Different letters indicate significant differences (p < 0.05) between groups.

In inguinal adipose tissue, relative expression of the PGC-1α gene did not show a significant change in any experimental group. NRF1 expression showed a significant effect in the cold exposure (p = 0.011) and exercise (p = 0.01) groups. There was significant interaction between exercise and cold exposure (p = 0.000), which means exercised in neutral temperature animals had a higher NRF1 expression than the control, cold immersed, and exercised in cold water groups. Tfam mRNA expression showed significant effects in the exercise group (p=0.000) and interaction (p=0.001), which means animals exercised in neutral temperature water had a higher Tfam expression than the control, cold immersed, and exercised in cold water animals. Post-hoc analyses showed Tfam expression was also higher in the cold immersion and exercised in cold water groups than the control (p = 0.014 and p = 0.004, respectively). Contrary to expectation, cold exposure had no effect on the relative expression of PGC-1α or Tfam (p=0.176 and p=0.475, respectively). 

**Figure 6.**
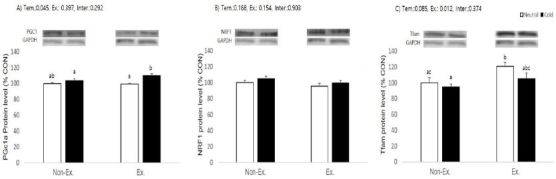
Effects of exercise or/and cold exposure on expression of mitochondrial biogenesis proteins in the soleus muscle. Data are presented as means ± SE (n=8 per group). (A) peroxisome proliferator-activated receptor gamma coactivator-1 alpha (PGC-1α); (B) nuclear respiratory factor 1 (NRF1); and (C) mitochondrial transcription factor A (Tfam) relative to the expression level of glyceraldehyde 3-phosphate dehydrogenase (GAPDH). Two-way ANOVA results: (A) temperature: 0.045, exercise: 0.397, interaction 0.292 (B) temperature: 0.168, exercise: 0.154, interaction 0.903 (C) temperature: 0.085, exercise: 0.012, interaction 0.374. Different letters indicate significant differences (p < 0.05) between groups.

### Protein level

To extend our observations at the gene level, we characterized the effects of exercise or/and cold exposure on the protein changes for PGC-1α, NRF1, and Tfam in type I fiber-rich soleus muscle. Because protein is expressed at a low level in white adipocytes, we could not test its protein expression. Protein expression was determined between tissues and relative protein band density values were calculated as the ratio of protein of interest to that of GAPDH. The data in the control group have been set to 100% for each tissue. 

Only temperature had a significant effect on PGC-1α protein expression (p=0.045), and neither exercise nor the interaction were significant (p = 0.397 and p = 0.292, respectively). Post-hoc analyses showed animals exercised in cold water had a higher PGC-1α expression than control and exercised in neutral temperature animals. NRF1 did not show significant changes in any experimental treatment. Tfam protein expression only showed significant effects for exercise (p=0.012), and neither temperature nor the interaction were significant (p = 0.085 and p = 0.374, respectively). 

## DISCUSSION

Studies show that individuals with metabolic disorders have reduced expression of mitochondrial genes in skeletal muscle and adipose tissue^15-19^. Given the potentially important role of mitochondrial dysfunction in the pathogenesis of numerous diseases, the stimulation of mitochondrial biogenesis has become an attractive therapeutic target^6, 22^. In this regard, despite the well-recognized link between mitochondrial biogenesis and stimuli such as exercise and cold exposure^23^, the synergy or additional effects of these stimuli are not well known. We determined temperature differences during exercise were the main contributor to the changes in mitochondrial biogenesis-related gene expression and proteins. Thus, a long-term goal of this line of research is to develop novel temperature-optimized training protocols in order to enhance performance, prevent several metabolic disorders, and/or treat diseases such as metabolic diseases, neurodegenerative diseases, and cardiovascular diseases. 

In the present study, the lack of body weight change during the 8 weeks of cold exposure is consistent with other studies that demonstrate no change in body weight following similar, prolonged periods of cold exposure^24,-27^. However, it is noteworthy that the cold exposed mice remained lighter than the control mice at all time points, despite displaying higher food intake than their counterparts at neutral temperature. Furthermore, studies have observed contradictory effects on body weight and white adipose tissue weight, with some studies showing a decreased weight gain in rodents exposed to cold and others showing no alterations in body weight and body fat composition^24, 25, 28, 29^. The discordance is likely multifactorial and may include differences in species and experimental protocols. However, we found that there was no difference in body weight for the cold immersed or exercised in cold groups compared with the control group. Only mice exercised in normal temperatures had significantly lower body weight. Moreover, soleus muscle weight was decreased in all experimental groups and inguinal adipose weight was decreased only in animals exercised in cold temperature water. 

In the mitochondrial biogenesis-related gene expression data presented, exercise and cold exposure interact in soleus muscle. Interestingly, the group differences show mice that exercised regularly had higher exercise-induced muscular mitochondrial biogenesis-related gene expression in colder environments than sedentary mice, and temperature induced mitochondrial biogenesis-related genes expression was also greater in exercised mice. In this regard, electromyogram (EMG) activities in the soleus muscles are increased by cold exposure in rats^30^. Such an enhanced EMG activity in the soleus muscle in response to cold temperature may be related to shivering-related metabolic activity. Slow motor units are primarily recruited during the shivering response following exposure to acute cold temperatures^31,32^. Exercise or cold alone had a partial effect on mitochondrial transcriptional regulators and caused only a limited increase in mitochondrial biogenesis-related protein expression. Regular exercise is a prerequisite for increases in muscle PGC-1α expression in response to cold. This role of exercise is emphasized by the fact that NRF-1 showed similar expression patterns as PGC-1α in response to exercise. Although our results did not show any changes in Tfam expression, PGC-1α facilitates the interaction between NRF-1 and Tfam, which regulates mitochondrial DNA replication and transcription. These findings are consistent with a previous report, which demonstrated that cold exposure is effective in increasing PGC-1α in soleus muscle from aged rats, whereas exercise alone was unable to produce an increase in PGC-1α expression^7^. Furthermore, O’Brien (2011)^8^ and Ihsan et al. (2015)^33^ demonstrated that cold-water immersion combined with aerobic exercise induces additive effects on the expression of PGC- 1α and other transcriptions factors of mitochondrial biogenesis. Slivka et al. (2012)^9^ has shown that post-exercise recovery for 3 hours in a cold environment can lead to higher levels of expression for genes implicated in mitochondrial biogenesis, such as PGC-1α. Furthermore, cold exposure alone may not be practical for actual application, because most people would prefer to be at a more comfortable temperature when recovering and would not have an additional 3 hours to dedicate to their rehabilitation or training. Therefore, this study will contribute to the field by determining the optimal and realistic stimuli needed for enhancing mitochondrial biogenesis. 

The current data does not demonstrate whether cold exposure is a necessary stimulus for induction of mitochondrial biogenesis-related genes expression in inguinal adipose tissue, in contrast to skeletal muscle. This data is very interesting, because adipose tissue directly senses temperature to activate thermogenesis. A possible interpretation of our results is that if thermogenic responses to cold in adipose tissue are dependent on a behavioral trigger such as exercise, the lack of a cold exposed effect in exercised mice would result from a muted response to cold in adipose tissue via shivering thermogenesis from muscle. Secondly, we did not aim to push mice to their physiological limits as is often done when testing thermogenic capacity, rather, our data have important implications for practical applications. Klingenspor (2003)^34^ and Cannon & Nedergaard (2004)^35^ show that extreme cold exposure will stimulate uncoupling proteins and oxidative capacity in the absence of physical activity. It suggests a threshold of activation below which exercise becomes relatively unimportant for thermogenesis in adipose tissue. However, in contrast to skeletal muscle, little is known regarding the specific mechanisms that may trigger exercise-induced increases in PGC-1α mRNA expression and mitochondrial biogenesis in adipose tissue. One possible explanation is the recently identified hormone regulated by PGC-1α, irisin, which is secreted from muscle into blood, activates thermogenic function in adipose tissues^35^. In adipose tissue, exercise did not increase the weight of inguinal adipose tissue, but increased mitochondrial biogenesis-related gene expression. It is unknown if exercise-induced inguinal adipocyte activation would do the same, either by a non-specific increase in metabolic demand, or via specific hormones, analogous to irisin from muscle or adipocytokines^25^. In addition, it seems difﬁcult to evaluate and compare the effects of exercise because of complicating factors such as type, duration, and intensity of physical activities, housing type, and feeding of the animals, which lead to different conclusions in these studies. 

In soleus muscle, only temperature had a significant effect on PGC-1α protein expression and only exercise had a significant effect on NRF1 protein expression. These differences in exercise- and/or cold-induced changes in protein profiles highlight the potential for discordance between gene and protein expression and further reinforce the notion that exercise and/or cold induce tissue remodeling. Consequently, our data demonstrate that exercise and/or cold exposure induces the gene expression of mitochondrial biogenesis markers in muscle and adipose tissues; however, the expression of these markers and the magnitude of the changes differed between the tissues and experimental treatments. 

## CONCLUSION

Our data demonstrate that exercise and/or cold exposure induces the gene expression of mitochondrial biogenesis markers in muscle and adipose tissues; however, the relative expression of these markers and the magnitude of the changes differed across the tissues and experimental treatments. Exercise and cold exposure increase the expression of mitochondrial biogenesis-related genes in soleus muscle. Only cold exposure had a significant effect on PGC-1α protein expression and only exercise had a significant effect on NRF1 protein expression. In inguinal adipose tissue, there was an interaction between exercise and cold exposure for the expression of mitochondrial biogenesis-related genes, but interestingly, cold exposure alone had no effect. Thus, we showed that exercise is a necessary condition for increasing mitochondrial biogenesis in inguinal adipose tissue. 

## LIMITATION

The current data imply only mRNA transcription with these interventions and are limited to this response since no protein in adipose tissue, mitochondrial function, browning of white fat, or whole body energy expenditure were measured. Future research should build off these data to focus on the effects of cold exposure during exercise on more functional aspects of mitochondrial development including protein accumulation, mitochondrial function, browning of white fat, and whole body energy expenditure. 
